# Quality of life of inguinal hernia patients in Taiwan: The application of the hernia-specific quality of life assessment instrument

**DOI:** 10.1371/journal.pone.0183138

**Published:** 2017-08-17

**Authors:** Chi-Cheng Huang, Feng-Chuan Tai, Tzung-Hsin Chou, Heng-Hui Lien, Jaan-Yeh Jeng, Thien-Fiew Ho, Ching-Shui Huang

**Affiliations:** 1 Department of Surgery, Cathay General Hospital SiJhih, New Taipei City, Taiwan; 2 School of Medicine, College of Medicine, Fu-Jen Catholic University, New Taipei City, Taiwan; 3 School of Medicine, College of Medicine, Taipei Medical University, Taipei City, Taiwan; 4 Department of Surgery, Cathay General Hospital, Taipei City, Taiwan; 5 Department of Surgery, National Taiwan University Hospital, Taipei City, Taiwan; Cardiff University, UNITED KINGDOM

## Abstract

**Background:**

With the development of prosthetic mesh and tension free techniques, the recurrence rate following inguinal hernia repair has been reduced, and hernia outcomes research should focus on post-operative quality of life and potential complications.

**Study design:**

A novel hernia quality of life assessment instrument, HERQL, was developed. The HERQL questionnaire comprises a 4-item summative pain score measuring pain and discomfort resulting from various strenuous activities. Symptomatic and functional domains, as well as post-operative satisfaction are evaluated as well.

**Results:**

A total of 386 HERQL surveys were completed by 183 patients with inguinal hernias. Internal consistency reliability of the summative pain score was satisfactory, with a Cronbach’s alpha of 0.85. Criterion validity was examined by concomitant assessment of the pain/discomfort and health impact subscales of the EQ-5D questionnaire, with substantial to moderate correlations. Pre-operative patients reported more severe hernia protrusion, more pain during mild to heavy exercise, and worse activity restriction and health impairment than the follow-up patients, indicating clinical validity. The conceptual structure of the HERQL demostrated the causal relationship between the formative symptomatic subscales and the reflective functional status indicators. Repeated measurement of the summative pain scores revealed an estimated time effect of -1.63, which was the rate of change in the summative pain score across the pre-operative, immediately post-operative, and follow-up 3-month periods suggesting the clinical responsiveness of the HERQL.

**Conclusions:**

This study will facilitate inguinal hernia outcomes research and enhance the quality of care for this common disease by providing a validated HERQL instrument with enhanced sensitivity.

## Introduction

Hernia repair is one of the most common procedures performed by surgeons worldwide. With the advancements in prosthetic mesh and improvements in tension free repair, the recurrence rate following hernia repair has been reduced dramatically, and hernia outcomes research should focus on post-operative quality of life and potential complications such as chronic non-disabling pain [1–7].

Quality of life research in hernia patients usually utilizes generic instruments, which reduces the measurement sensitivity and limits the clinical applications. Indeed, Jensen et al. [8] have noted the need for standardization in the measurement of quality of life after hernia repair, and an internet-based registry for post-surgery outcomes research for various hernia types has been advocated [9].

Some hernia-specific outcomes research instruments have been published. The Carolinas Comfort Scale (CCS) was developed for patients undergoing mesh repairs and was validated in abdominal wall/groin hernias with both laparoscopic and open repairs [10], and a preference for the CCS rather than the generic SF-36 was reported. Site-specific instrument such as the COMI-hernia (Core Outcome Measures Index adapted for hernia), which was a groin hernia-specific measuring tool, has been reported [11]. An effective and validated hernia-specific assessment instrument for outcomes research has thus become an urgent need in surgical communities.

The aim of the study was to provide a validated quality of life assessment tool, HERQL, to facilitate hernia outcomes research. In Taiwan, the National Health Insurance (NHI), which is the only health care system, enrolls 97% of all of the country’s medical providers and about 99% of the population [12]. All inguinal hernia repair surgeries are compensated according to the Tw-DRGs (Taiwan Diagnosis Related Groups) while most mesh devices, especially those commercialized recently, have to be paid at private expense due to the limited NHI budget. Therefore, there are gaps in the coverage and applicability of existing hernia quality of life instruments developed in Western countries. Initially, the HERQL was designed to deal with both inguinal and abdominal wall hernia while in current study only inguinal hernia was addressed. The usability, validity and reliability of the HERQL as well as the application for Taiwanese inguinal hernia patients were performed and reported in current study.

## Materials and methods

The study has been reviewed and approved by the Institute Review Board of Cathay General Hospital under the access number CGH-P102069. The purpose and procedures of the study were explained by the primary investigator (CSH), and written informed consent was obtained from all participants.

### A. The conceptualization and pilot study of the HERQL

Initially a quality of life measuring instrument for both inguinal and abdominal wall hernia was proposed, encompassing symptomatic and functional domains experienced by patients with diverse hernia types. Due to the limited cases of abdominal wall hernia and the concern of anatomical discrepancy, only inguinal hernia was addressed in the current study. Here we presented the validation of the HERQL for inguinal hernia in Taiwan, and the applicability of the HERQL for abdominal wall hernia with possible modifications will be evaluated separately.

The content and dimensions of the HERQL were conceptualized from literature reviews and through panel discussions of the Taiwan Hernia Society (THS). Published hernia-specific instruments including the Activities Assessment Scale (AAS), CCS, COMI-hernia, Brief Pain Inventory (BPI), and Inguinal Pain Questionnaire (IPQ) were consulted [10–11, 13–15]. All referred questionnaires were compared and appraised systematically (S1 Table), and questionnaire items coinciding with the goals of developing the HERQL were retrieved. Candidate items are detailed in S2 Table and were categorized into four a priori domains, namely symptom, function, operation-related, and patient satisfaction.

These candidate items were evaluated in the pilot study with 30 pre-operative inguinal hernia patients, with interviews to assess what is important to these patients among these questions, using a five-point rating scale (from the most relevant to not relevant at all). Three operation-related items and eventration were discarded since these items were recognized as irrelevant by hernia patients (S2 Table). Mutually exclusive questions were stacked together while the immediate pain severity and the global outcome/treatment satisfaction were subdivided into more questionnaire items to enhance sensitivity. S2 Table showed the relationships between the candidates from literature reviews and the corresponding HERQL scales. It deserves notice that hernia protrusion, analgesic usage, and hernia’s impact on health were the HERQL-specific scales and were included in the pilot study as well. The ideal questionnaire length was determined to be no more than 20 items to encourage acceptance after referring to the questionnaire length among published works (S1 Table).

Successive explorative factor analyses were performed for the preliminary HERQL to identify any underlying higher-order construct (factor). The Principal component analysis showed that two components explained 57.5% of the variance of the HERQL response and a common factor analysis with the oblique PROMAX rotation further augmented the existence of the two common factors with wide separations of symptomatic and functional variables (S1 Fig). It was anticipated that, based on the results of the pilot study, albeit with a limited sample size and compromised statistic power, did point out that the symptomatic and functional domains would be retained in the final version of the HERQL, whereas the operation-related and patient satisfaction domains were potentially expendable. More details are available in the Supporting Information.

### B. Study population

Inclusion criteria were patients who planned to or had undergone inguinal hernia repairs at Cathay General Hospital, a tertiary referral institute in Metropolitan Taipei. Both pre-operative and post-operative hernia patients were enrolled. Exclusion criteria were concurrent participation in another human subject study, mental problems with difficulties understanding and completing the questionnaire, and refusal. Demographic characteristics, operation details, and follow-up events were retrieved from chart reviews.

### C. Field study, validation and the conceptual structure of the HERQL

The HERQL was validated in a large-scale field study with the target sample size of 350 completed questionnaires. Enrolled subjects were asked to fill out the EQ-5D-5L, a brief and generic quality of life instrument, concurrently for the purpose of criterion validation [16] (Supplementary methods). The correlations of mobility, usual activities, and pain/discomfort scores of the EQ-5D-5L from the same patient with the corresponding scores of the HERQL (activity restriction, health impact, and pain upon various strenuous activities) were evaluated.

Internal consistency reliability was evaluated for multi-item subscales, and was defined as a Cronbach’s alpha coefficient greater than 0.70 [17]. Clinical validity was evaluated by known-group comparisons, which were conducted by comparing patients with stable conditions (post-operative hernia patients without observable complications and with a minimum follow-up time of three months) and those under active treatment (pre-operative patients), under the hypothesis that pre-operative patients might have higher symptomatic scores and lower functional scores. Since quality of life scores were not always distributed normally, the Wilcoxon rank sum test was used for between-group comparisons. All tests were two-sided, and the Bonferroni correction with a reduced α level of 0.01 was applied for multiple comparisons.

The clinical responsiveness of the HERQL was evaluated through repeated measurements within the same patient. Only pre-operative patients were proceeded with repeated HERQL surveys, and were re-assessed at predefined time intervals (one week and three months post-operatively). The unconditional linear growth model was used to adjust for clustering of repeated measures on the same individual (Supplementary Methods). Fluctuations of quality of life scores in coherence with distinct clinical scenarios of hernia, such as active treatment (pre-operative), immediately post-operative, and post-operative three-month follow-up, suggested adequate responsiveness of the HERQL.

In addition to the original multi-item and single-item subscales, higher-order constructs designed to capture the correlations between latent factors for the HERQL were elaborated with the aid of structural equation modeling (SEM) and more statistic details could be found in the Supplementary Methods [18].

## Results

### A. Enrolled inguinal hernia patients

A total of 386 successful HERQL assessments (140 pre-operative, 158 immediately post-operative, and 88 post-operative with a minimum follow-up of three months) were performed in 183 inguinal hernia patients, each with 1 to 3 assessments during the study period between Aug 1, 2014 and Sep 30, 2015. Among these patients, 130 had both the pre-operative and post-operative surveys, including 69 with the immediately post-operative surveys (within one week), 56 with the immediately post-operative and post-operative 3-month surveys. The demographic and clinical features of the surveyed population are detailed in Table 1.

**Table 1:** Demographic and clinical features of enrolled patients.

	Inguinal hernia (N = 183)
Sex (M:F)	163:20
Age, mean (SD)	62.2 (15.3)
Body mass index	23.7 (3.1)
Groin hernia (unilateral:bilateral)	137:46
Recurrent hernia	31 (16.9%)
Incarcerated hernia	22 (12%)
Complications	
Seroma	6(3.3%)
Hematoma	7(3.8%)
Wound infection	4(2.2%)
Chronic pain	12(6.6%)
Urine retention	5(2.7%)
Sepsis/fever	1(0.6%)

During the enrollment period, most hernias were repaired with the open approach, except for 17 laparoscopic totally extraperitoneal (TEP) and 5 laparoscopic transabdominal preperitoneal (TAPP) for 7 unilateral/15 bilateral inguinal hernia repairs. The mesh devices incorporated included Perfix^™^ Plug and Perfix^™^ Light Plug (Bard, Murray Hill, New Jersey, US); PROLENE^™^ Polypropylene Hernia System and ULTRAPRO^™^ Hernia System (Ethicon, Somerville, New Jersey, US); KUGEL^™^ Hernia Patch, and Modified KUGEL^™^ Hernia Patch (Bard, Murray Hill, New Jersey, US). For laparoscopic repairs, 3DMAX^™^ and COMPOSIX^™^ L/P were used with the Sorbafix^™^ Absorbable Fixation System (Bard, Murray Hill, New Jersey, US) for mesh fixation.

### B. Determination of the HERQL content

The revised HERQL questionnaire from the pilot study includes a 4-item summative pain score (0–10 point Likert-type scale in each item) measuring pain and/or discomfort resulting from rest, mild, moderate, or heavy activities (Q01, Q03, Q04, and Q05). The summative pain score was the additive sum of these four items (range: 0~40). Activity restriction due to hernia-induced pain or discomfort was evaluated with another 0–10 point Likert-type scale (Q09). Items categorized into the symptomatic and functional domains measured with 5-point Likert-type scales, included hernia protrusion (Q02), analgesic use for hernia (Q08), hernia’s impact on health (Q11), economic burden (Q12), and subjective quality of life/global health perception (Q13), with higher values indicating more compromised functional status or worse symptom burden. Three questionnaire items were designed with checkboxes displaying multiple selections. They were discomfort in the last week (Q10), conditions most affected by hernia (Q06), and restricted activities due to hernia (Q07).

Six auxiliary items were used for the post-operative module (post-operarative patient satisfactory domain), including one with multiple selections for potential complications following hernia repairs (Q16) and five items measuring mesh foreign body sensation (Q15), severity of complications (Q17), overall satisfaction for hernia repair (Q18), confidence that hernia will not recur (Q19), and quality-of-life improvement by hernia repair (Q20). All except Q16 were arranged as 5-point Likert-type scales, with higher values representing more compromised functionality or worse symptoms. The complete HERQL instrument is displayed in the Supporting Information.

### C. Internal consistency reliability, criterion and clinical validity

Table 2 shows the distributions of the HERQL scores in the field study (n = 386), while 246 of them were also surveyed with the post-operative auxiliary module.

**Table 2:** Distributions of the HERQL scores with Likert’s type scales in the field study (n = 386).

Item	Possible range of scores	N	Mean	Standard deviation	Minimum (percentage)	Maximum (percentage)
Q01	0–10	385	0.8	1.9	0(78.4%)	10(0.3%)
Q02	1–5	384	2.3	1.7	1(60.2%)	5(21.9%)
Q03	0–10	385	1.5	2.4	0(63.1%)	10(0.8%)
Q04	0–10	385	1.2	2.3	0(70.1%)	10(0.3%)
Q05	0–10	383	1.2	2.4	0(74.4%)	10(1%)
Q08	1–5	384	2.0	1.5	1(63.3%)	5(14.6%)
Q09	0–10	383	2.3	2.9	0(49.1%)	10(1.6%)
Q11	1–5	384	1.6	0.8	1(58.9%)	4(3.9%)
Q12	1–5	384	1.2	0.5	1(86.7%)	4(0.8%)
Q13	1–5	385	2.2	0.9	1(16.9%)	5(0.8%)
Q15	1–5	240*	1.7	1.1	1(67.5%)	5(2.9%)
Q17	1–5	240*	1.5	0.7	1(61.3%)	4(1.7%)
Q18	1–5	241*	1.4	0.6	1(60.6%)	4(0.4%)
Q19	1–5	241*	2.1	0.8	1(22%)	4(1.7%)
Q20	1–5	241*	1.8	0.9	1(48.1%)	5(0.4%)

*Post-operative auxiliary module was conducted in 246 surveys.

For the four items measuring the summative pain score (Q01, Q03, Q04 and Q05), the standardized Cronbach’s alpha coefficient was 0.85, indicating a satisfactory internal consistency reliability. The criterion validity was evidenced from the significant correlations (all P-values less than 0.0001) between the HERQL items assessing pain severity and the pain scores reported by the EQ-5D-5L. The correlation coefficients with the EQ-5D-5L pain score were 0.43, 0.58, 0.6, and 0.52 for Q01, Q03, Q04, and Q05, respectively. Items measuring hernia’s health impact (Q11) and activity restriction (Q09) were positively correlated with the corresponding EQ-5D-5L scores as well (0.42 for Q11 and 0.31 for Q09).

Clinical validity was examined by comparing the pre-operative surveys (n = 140) with the follow-up surveys (minimum follow-up of three months, n = 88). Significant differences were observed (Table 3) in Q03 (pain at mild activity), Q04 (pain at moderate activity), and Q05 (pain at heavy activity), as well as in Q02 (hernia protrusion), Q09 (activity restriction), Q11 (hernia’s impact on health), and Q13 (general health status). In general, post-operative patients reported less pain sensation, improved activity restriction, and less impact of hernia on their health following surgical repair.

**Table 3:** Known-group comparisons of the HERQL.

		Pre-operative surveys		Follow-up surveys	
		n = 140		n = 88	
		Mean	SD	Mean	SD
Q01	Pain at rest	0.84	2.02	0.43	1.51
Q02*	Protrusion	**4.07**	1.37	**1.23**	0.75
Q03*	Pain at mild activity	**2.06**	2.71	**0.51**	1.53
Q04*	Pain at moderate activity	**1.64**	2.53	**0.51**	1.68
Q05*	Pain at heavy activity	**2.14**	2.96	**0.4**	1.48
Q08	Analgesic usage	1.23	0.79	1.19	0.71
Q09*	Activity restriction	**2.71**	3.12	**1.05**	2.21
Q11*	Impact of hernia on health	**1.81**	0.86	**1.24**	0.63
Q12	Economic burden	1.19	0.54	1.07	0.3
Q13*	Global health	**2.37**	0.96	**1.77**	0.71

* Questionnaire items with a significant difference between groups (P<0.01). Between-group comparisons were analyzed with non-parametric Wilcoxon tests.

### D. The conceptual structure of the HERQL

**Fig 1:**
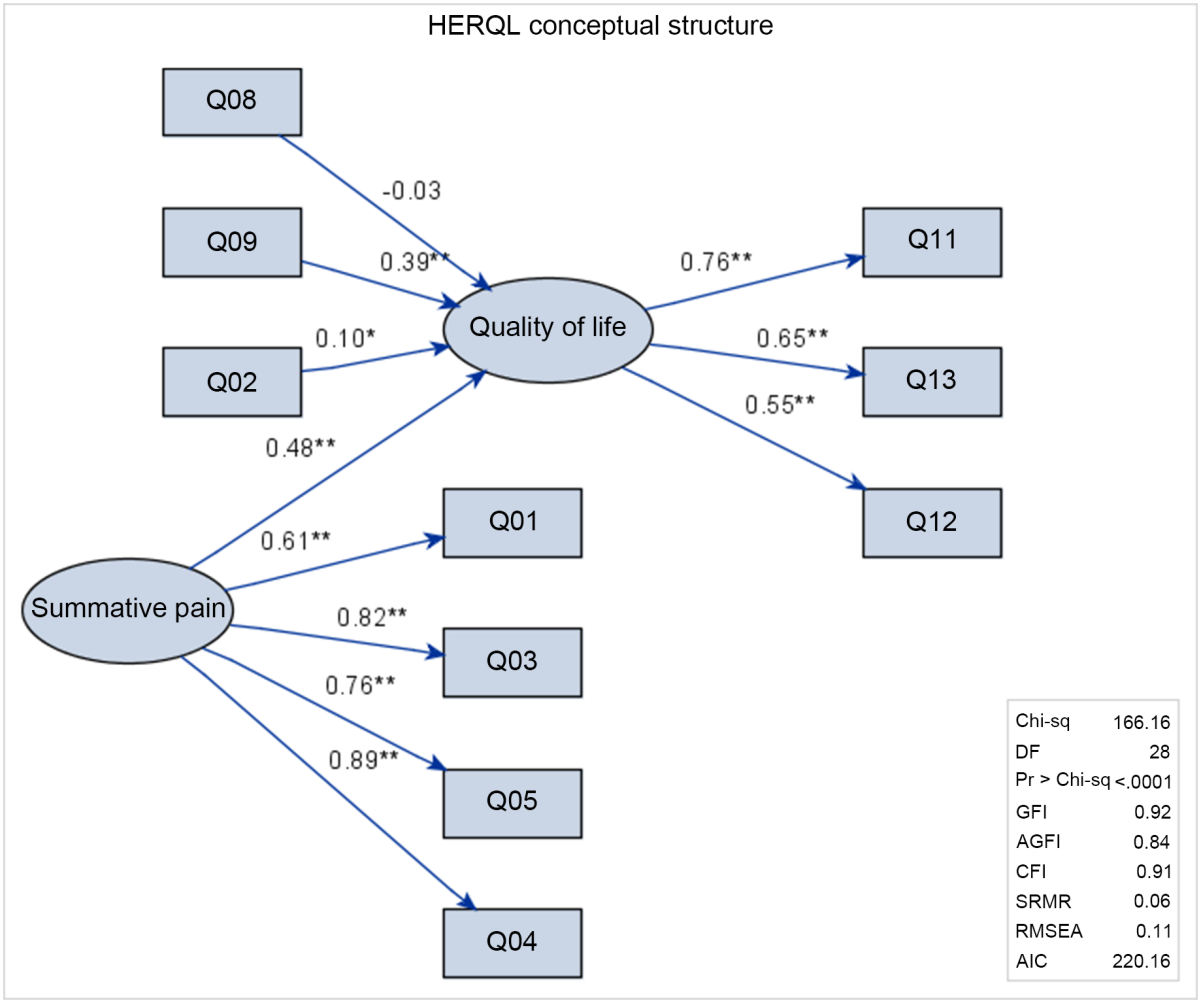
The conceptual structure of the HERQL. Circles: latent factors, rectangles: measured variables (questionnaire items). Q01: pain at rest, Q02: hernia protrusion, Q03: pain from mild activity, Q04: pain from moderate activity, Q05: pain from heavy activity, Q08: analgesic usage, Q09: activity restriction, Q11: hernia’s impact on health, Q12: economic burden, Q13: quality of life/global health. Arrows indicate the direction of regressive relationships. Numeric values are regression weights. *P<0.05, **P<0.01.

Fig 1 depicts the conceptual structure of the HERQL with all Likert-type scale items incorporated. GFI (goodness of fix index), AGFI (adjusted GFI), CFI (comparative fit index), and SRMR (standardized root mean square residual) suggested a good model fit, while RMSEA (root mean square error of approximation) was slightly compromised. The latent pain factor was manifested by four indicator variables (Q01, Q03-Q05), with all regression weights being significant. The regression weight from the latent pain to the latent quality of life factor was 0.48 and was significant at P<0.01, meaning that a higher subjective pain sensation impaired quality of life. Other causative (formative) symptoms were hernia protrusion (Q02), analgesic usage (Q08), and activity restriction (Q09), and only analgesic usage was not a significant predictor for latent quality of life status (regression weight: 0.1 for hernia protrusion, P<0.05, and 0.39 for activity restriction, P<0.01). Three functional domain items, hernia’s impact on health, economic burden, and quality of life/global health perception (Q11-Q13), were indicators (reflective variables) for the latent quality of life, and all were highly significant at P<0.01.

**Fig 2:**
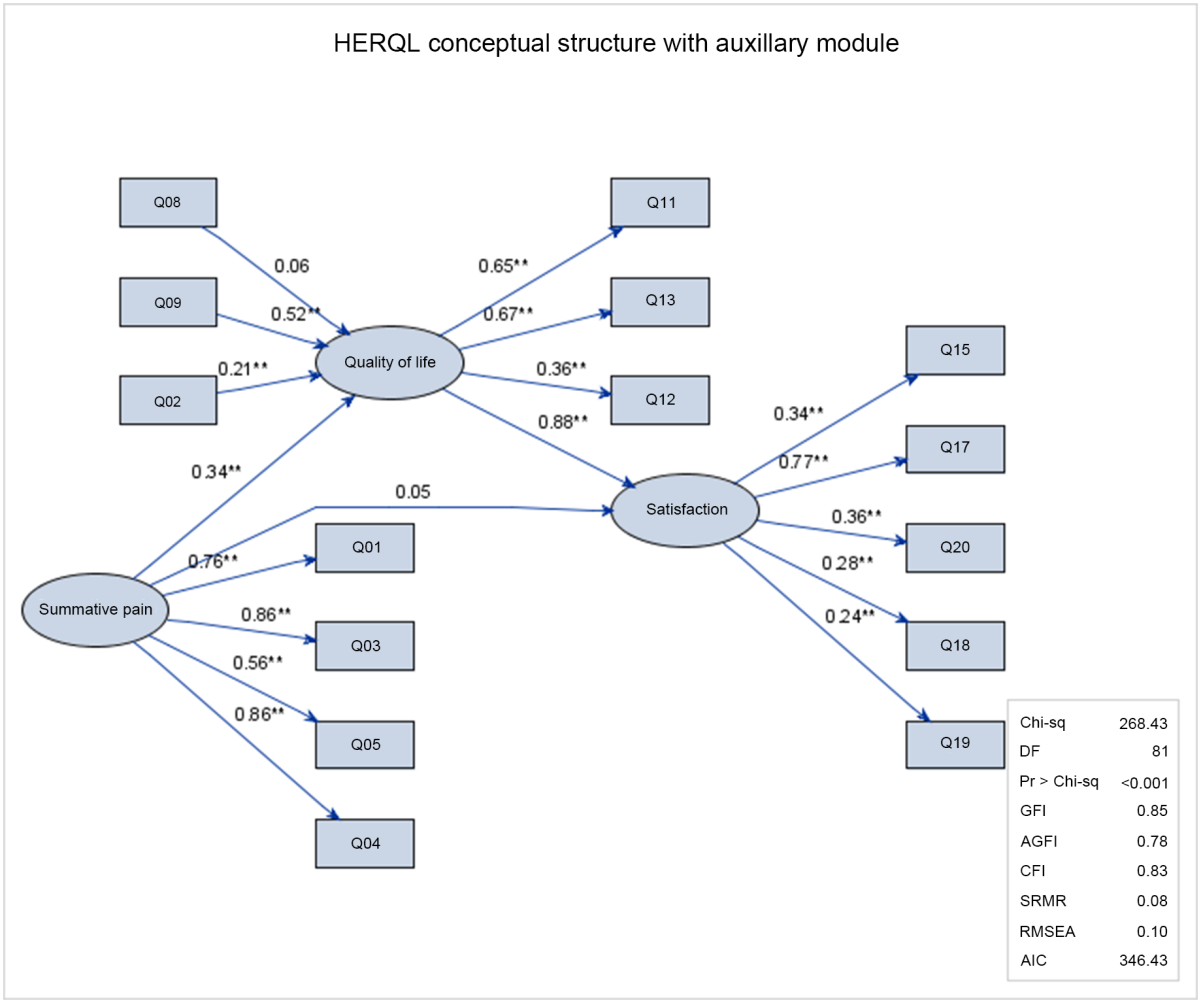
The conceptual structure of the extended model of the HERQL with the auxiliary post-operative patient satisfaction module. Circles: latent factors, rectangles: measured variables (questionnaire items). Q01-05, Q08-09, and Q11-13 are the same as in Fig 1. Q15: foreign body sensation, Q17: complication severity, Q18: overall satisfaction, Q19: confidence in hernia repair, Q20: quality of life improvement by hernia repair. Arrows indicate the direction of regressive relationships. Numeric values are regression weights. *P<0.05, **P<0.01.

Fig 2 depicts the extended HERQL conceptual structure with additional five auxiliary items for the post-operative patient satisfaction domain. Compared with the original model, this extended model suffered from slightly compromised model fit indices, which might be resulted from the more complicated structure. Among the five post-operative items, mesh foreign body sensation (Q15), complication severity (Q17), satisfaction with hernia repair (Q18), confidence in hernia repair (Q19), and quality of life improvement by hernia repair (Q20) were significant indicators of the underlying patient satisfaction (P<0.01). The latent pain factor was predictive of the latent quality of life status, with a significant and positive regression weight of 0.34 (P<0.01). The latent quality of life status was predictive of the post-operative patient satisfaction factor (regression weight: 0.88, P<0.01), indicating that worse quality of life perception predicted compromised satisfaction. The direct effect of the summative pain upon the patient satisfaction became insignificant given the effect of the latent quality of life factor.

### E. Clinical responsiveness

**Fig 3:**
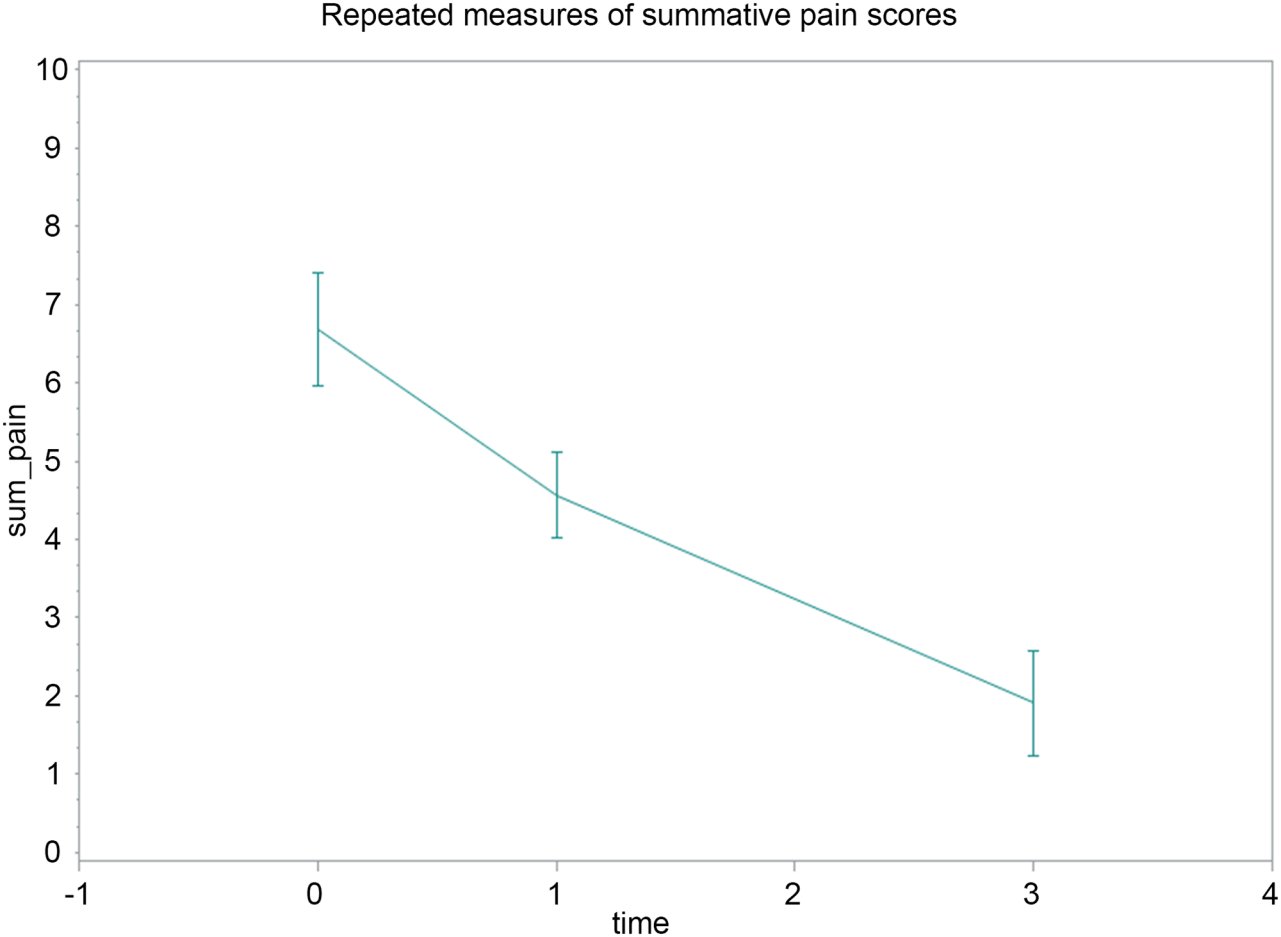
Repeated measures of the summative pain scores. Mean and 95% confidence interval of the summative pain scores are depicted at three time points. Vertical axis: summative pain score, horizontal axis: pre-operative (time = 0), immediately post-operative (time = 1), and post-operative 3-month of interval (time = 3).

The 367 HERQL summative pain scores measured during the pre-operative, immediately post-operative, and post-operative three-month period from the 175 inguinal hernia patients were evaluated for clinical responsiveness. The unconditional linear growth model was used, and the structure of the variance and covariance matrices within individuals was explored by experimental data. An unstructured variance/covariance matrix was determined from model fit statistics (S3 Table). The estimate of the intercept was 6.68 (P<0.001), which was the initial (pre-operative) value of the summative pain score. The estimate of the slope was -1.63 (P<0.001), which was the rate of change in the summative pain score across repeated measures. The results support the conclusion that surgical intervention improved hernia-associated pain, and the effect was noticeable in the immediately post-operative period and persisted through three months after hernia repair (Fig 3).

## Discussion

As one of the most common surgically treatable diseases, inguinal hernia enjoys reduced recurrence because of the development of prosthetic mesh and tension-free techniques [1–7]. Consequently, hernia outcomes research should be tailored to improving post-operative quality of life and eliminating potential complications such as chronic non-disabling pain.

Indeed, impairment in quality of life is a major reason why hernia patients seek surgical repairs [10]. In addition to conventional clinical outcomes such as recurrence, how patients perceive the surgical intervention and how they evaluate the efficacy of hernia repair will be revealed by quality of life assessment.

During the development of the HERQL, extensive literature reviews were performed to identify studies dealing with hernia repair outcomes [10–11, 13–15]. The CCS was developed specifically for patients undergoing hernia repairs with prosthetics under the hypothesis that the placement of mesh might result in long-term physical and mental impairment [10]. The CCS is a 23-item questionnaire that measures severity of pain, sensation, and movement limitations from the mesh in 8 categories of movement, and has been applied for both laparoscopic and open abdominal wall hernia repairs with ePTFE mesh (Gore-Tex Dualmesh^®^, W.L. Gore & Associates, Flagstaff, Arizona, US) as well as for TAPP, TEP, and modified Lichtenstein groin hernia repairs [19–20]. In one study, the CCS was discriminative between the Surgipro^™^ (Covidien, Mansfield, Massachusetts, US) and Progrip^™^ (Parietene Progrip^™^, Sofradim Production, Trévoux, France) mesh in open groin hernia repairs [21]. The major drawback of the CCS is that it cannot be used pre-operatively.

The inguinal hernia-specific instrument, COMI-hernia, was designed with a single item each for the domains of pain, function, symptom-specific well-being, general quality of life, and social and work disability pertaining to global treatment outcome, satisfaction, and patient-rated complications [11]. Completion rates were high due to its brevity. Two pain-focused instruments were the BPI and the IPQ, with the former designed for pre-operative assessments of both abdominal wall and inguinal hernias, and the latter for post-operative surveys and limited to inguinal hernia [14–15]. The AAS was targeted for groin hernia repairs with both laparoscopic and open repair approaches measuring limitations in sedentary, ambulatory, work and exercise activities [13].

The internal consistency reliability of the summative pain score and the clinical validity of the HERQL comparing patients under active treatment and those at the post-operative three-month follow-up was ascertained. With 4 items measuring severity of pain under various strenuous activities, and considering that pain is a deterministic element in hernia disease, a single latent pain factor (summative pain score) was constructed to be independent of other symptomatic measures. Other symptomatic domain items were formative and were postulated to have a causal effect upon the latent quality of life factor. All functional domain items were reflective of the underlying latent quality of life factor, and the post-operative module items were reflective of the underlying patient satisfaction factor.

Initial efforts to use SEM for quality of life measurement can be traced back to Fayers et al. [22], who aimed at separating the causal variables (symptoms) from the effect indicator variables (functional domains). The causal and indicator variables model forms the basis of the conceptual structure of the HERQL [23–24]. The critical theory underpinning the causal-indicative duality is that hernia-associated symptoms may impair subjective quality of life perception, which is subsequently reflected in the functional domains and indicator variables, as well as in patient satisfaction from the postoperative group. Figs 1 and 2 show the relational diagrams of the original and extended models, indicating the feasibility of SEM approach for the HERQL.

The clinical responsiveness of the HERQL was demonstrated by repeated measures of the summative pain scores across the pre-operative, immediately post-operative, and post-operative three-month surveys. It is understood that quality of life is a function of time, and the fluctuations of subjective quality of life scores corresponding to clinical scenarios supported the conclusion that HERQL was responsive to changes over time with adequate sensitivity. Since enrolled patients were assessed with an unequal number of repeated measurements, we used an unconditional growth (mixed) model to overcome the problem of missing values, to prevent list-wise deletion of all measures from patients with at least one missing value among successive surveys. Within subject correlations (repeated measures of the same participant) were considered as well. Our results showed that progressively less pain was reported when patients underwent hernia repairs and were stable at follow-up, as compared with the initial untreated disease, demonstrating that patients reported less and less pain and discomfort over time following hernia repair (Fig 3).

Our previous studies have ascertained the efficacy of tension-free hernia repair, which has a low recurrence rate and provides early ambulation [25–27]. Low failure rates have made “hard” end points, such as recurrence or wound complications, less pronounced, so the focus of hernia outcomes research should be directed to “soft” end points such as patients’ subjective perception and quality of life. The self-reported HERQL questionnaire provides such end points from the patient’s perspective.

There are some limitations of the current study. First, our modest sample size may have resulted in compromised statistical power. Second, we invited all inguinal hernia patients who had visited our clinics during the study period. Although best efforts had been tried, not every pre-operative participant could complete all the successive surveys. Our experience shows that loss of follow-up becomes a major problem for patients who had hernia repaired more than 3 months ago, and further quality of life surveys become illusive. To assess long-term results of hernia surgery, mailed questionnaires or mobile app to enhance the responsiveness may be conducted in future studies. Third, four questionnaire items (including one in the post-operative module) containing multiple-selection checkboxes were actually not addressed in the current study. Multiple-selection checkbox design did provide an efficient way to gather the maximum amount of information with limited questionnaire length. Future studies could take advantage of these assessments regarding recent co-morbidity (discomfort in the last week, Q10), conditions most affected by hernia (Q06), restricted activities due to hernia (Q07), and hernia repair complications (Q16).

We believe that our study developed a validated quality of life assessment tool to facilitate outcomes research for inguinal hernia and enhance the health care quality of hernia patients. The quantitative data gathered from the HERQL will provide invaluable evidence to reinforce resource allocations, preventive medicine, and even tailored approaches for personalized hernia repairs.

## Supporting information

S1 Table Comparisons among hernia quality of life surveys. (DOCX)

S2 Table Candidate items from literature reviews and the corresponding HERQL items and domains. (DOCX)

S3 Table Fit statistics of models with different variance-covariance structures. Fit statistics: -2 restricted log likelihood score (-2RLL), Akaike information criterion (AIC), finite-population corrected Akaike information criterion (AICC), Bayesian information criterion (BIC); smaller is better for AIC, AICC, and BIC.
(DOCX)

S1 Fig PROMAX-rotated factor loadings of the pilot study. Q01: pain at rest, Q02: hernia protrusion, Q03: pain from mild activity, Q04: pain from moderate activity, Q05: pain from heavy activity, Q08: analgesic usage, Q09: activity restriction, Q11: hernia’s impact on health, Q12: economic burden, Q13: quality of life/global health.
(TIF)

S1 File Pilot study. (DOCX)

S2 File Supplementary methods. (DOCX)

S3 File The HERQL questionnaire. (PDF)

S4 File Covariance matrix in excel dataset formats. (XLS)

S5 File Covariance matrix in SAS dataset formats. (SAS7BDAT)
