# Supplementary material for: Quality of life of inguinal hernia patients in Taiwan: The application of the hernia-specific quality of life assessment instrument
Source: PLoS One. 2017 Aug 17;12(8):e0183138. doi: 10.1371/journal.pone.0183138 (PMC5560705; doi:10.1371/journal.pone.0183138)
Supplement: S1 Table — (DOCX) [file pone.0183138.s001.docx]

**S1 Table. Comparisons among hernia quality of life surveys.**

| Instrument | Hernia type | Surgical procedure | Assessment timeframe | Domains | Items |
| --- | --- | --- | --- | --- | --- |
| Activities assessment scale | Groin | Laparoscopic/open | Pre-/post-operative | Sedentary, ambulatory, work and exercise activities | 11 |
|  |  |  |  |  |  |
| Carolinas comfort scale | Abdominal/groin | Laparoscopic/open | Post-operative | Pain, sensation, movement limitations from lying down, bending over, sitting up, daily activities of life, coughing or deep breathing, walking, stairs, and exercise | 23 |
|  |  |  |  |  |  |
| COMI-hernia | Groin | Unknown | Pre-/post-operative | Pain, function, symptom-speciﬁc well-being, general quality of life, social and work disability | 6(pre-operative) |
|  |  |  |  | Global treatment outcome, satisfaction with the treatment received, patient-rated complications and their bothersomeness | 6(post-operative) |
|  |  |  |  |  |  |
| Brief pain inventor | Abdominal/groin | Not applicable | Pre-operative | Pain severity/interference | 11 |
|  |  |  |  |  |  |
| Inguinal pain questionnaire | Groin | Open | Post-operative | Pain intensity, interference with daily activities | 18 |
|  |  |  |  |  |  |
