# Supplementary material for: Quality of life of inguinal hernia patients in Taiwan: The application of the hernia-specific quality of life assessment instrument
Source: PLoS One. 2017 Aug 17;12(8):e0183138. doi: 10.1371/journal.pone.0183138 (PMC5560705; doi:10.1371/journal.pone.0183138)
Supplement: S2 Table — (DOCX) [file pone.0183138.s002.docx]

**S2 Table. Candidate items from literature reviews and the corresponding HERQL items and domains.**

|  |  |  |  |  |  |  |  |
| --- | --- | --- | --- | --- | --- | --- | --- |
| Description of question | | | Domain |  | HERQL item(domain) | | |
| Sensation of mesh | |  | Symptom | | Q15 (post-operative satisfaction) | | |
| Severity of immediate pain | | | Symptom | | Q01,Q03,Q04,Q05 (pain) | | |
| Movement limitation | | | Symptom | | Q06,Q09 (symptom) | |  |
| Sex impairment | |  | Symptom | | Q10 (function) | |  |
| Interfering strenuous activity | | | Symptom | | Q07,Q09 (symptom) | |  |
| Interfering moderate activity | | | Symptom | | Q07,Q09 (symptom) | |  |
| Interfering walking/climbing stairs | | | Symptom | | Q07,Q09 (symptom) | |  |
| Low back pain | |  | Symptom | | Q10 (function) | |  |
| Gastro-intestinal dysfunction | | | Symptom | | Q10 (function) | |  |
| Micturition | |  | Symptom | | Q10 (function) | |  |
|  |  |  |  |  |  |  |  |
| Seroma formation | |  | Operation-related | |  |  |  |
| Hematoma formation | | | Operation-related | |  |  |  |
| Wound problem (infection) | | | Operation-related | |  |  |  |
|  | | |  | |  |  |  |
| Global outcome/satisfaction with treatment | | | Patient satisfaction | | Q18,Q19,Q20 (post-operative satisfaction) | | |
| Patients' rated complications | | | Patient satisfaction | | Q16 (post-operative satisfaction) | | |
| Bothersome of complications | | | Patient satisfaction | | Q17 (post-operative satisfaction) | | |
|  |  |  |  |  |  |  |  |
| Activities of daily life | | | Function |  | Q07,Q09 (symptom) | |  |
| Global quality of life | | | Function |  | Q13 (function) | |  |
| Chronic non-disabling pain | | | Function |  | Q16 (post-operative satisfaction) | | |
| Less accomplishment at work | | | Function |  | Q06,Q07 (symptom) | |  |
| Less accomplishment at home | | | Function |  | Q06,Q07 (symptom) | |  |
|  |  |  |  |  |  |  |  |
| Eventration | |  | Optional | |  |  |  |
| Financial difficulty | |  | Optional | | Q12 (function) | |  |
