# Supplementary material for: Quality of life of inguinal hernia patients in Taiwan: The application of the hernia-specific quality of life assessment instrument
Source: PLoS One. 2017 Aug 17;12(8):e0183138. doi: 10.1371/journal.pone.0183138 (PMC5560705; doi:10.1371/journal.pone.0183138)
Supplement: S3 Table — Fit statistics: -2 restricted log likelihood score (-2RLL), Akaike information criterion (AIC), finite-population corrected Akaike information criterion (AICC), Bayesian information criterion (BIC); smaller is better for AIC, AICC, and BIC. (DOCX) [file pone.0183138.s003.docx]

**S3 Table. Fit statistics of models with different variance-covariance structures.** Fit statistics: -2 restricted log likelihood score (-2RLL), Akaike information criterion (AIC), finite-population corrected Akaike information criterion (AICC), Bayesian information criterion (BIC); smaller is better for AIC, AICC, and BIC.

| Model |  | -2RLL | AIC | AICC | BIC |
| --- | --- | --- | --- | --- | --- |
| Compound symmetry | | 2497.9 | 2501.9 | 2502 | 2508.3 |
| Unstructured | | 2480.3 | 2486.3 | 2486.3 | 2495.8 |
| Autoregressive | | 2498.8 | 2502.8 | 2502.8 | 2509.1 |
| Autoregressive with heterogeneous variance | | Not converged | | | |
