# Supplementary figures and images for: Quality of life of inguinal hernia patients in Taiwan: The application of the hernia-specific quality of life assessment instrument
Source: PLoS One. 2017 Aug 17;12(8):e0183138. doi: 10.1371/journal.pone.0183138 (PMC5560705; doi:10.1371/journal.pone.0183138)

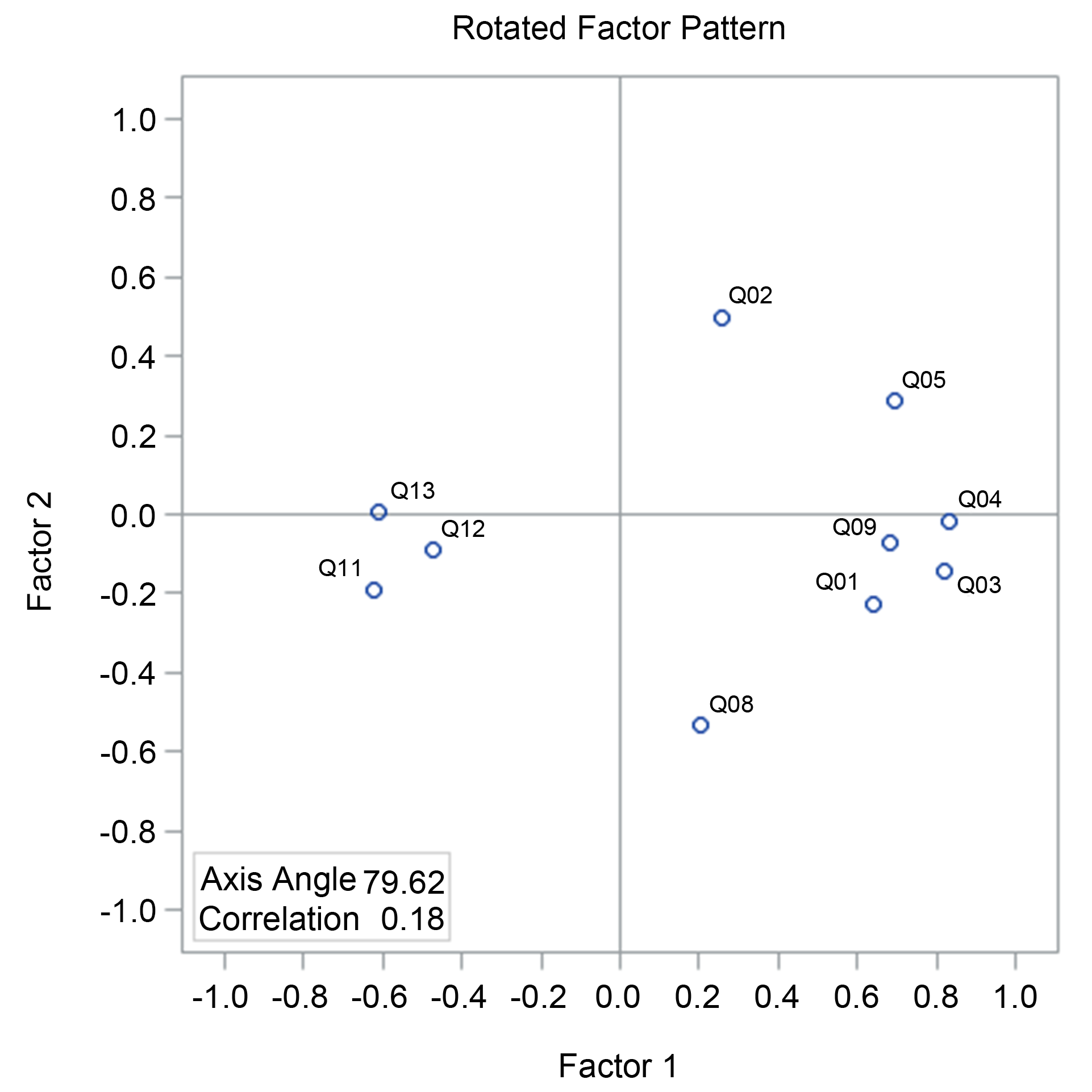

Supplement: S1 Fig — Q01: pain at rest, Q02: hernia protrusion, Q03: pain from mild activity, Q04: pain from moderate activity, Q05: pain from heavy activity, Q08: analgesic usage, Q09: activity restriction, Q11: hernia’s impact on health, Q12: economic burden, Q13: quality of life/global health. (TIF) [file pone.0183138.s004.tif]
