# Supplementary material for: Quality of life of inguinal hernia patients in Taiwan: The application of the hernia-specific quality of life assessment instrument
Source: PLoS One. 2017 Aug 17;12(8):e0183138. doi: 10.1371/journal.pone.0183138 (PMC5560705; doi:10.1371/journal.pone.0183138)
Supplement: S1 File — (DOCX) [file pone.0183138.s005.docx]

**S1 File. Pilot study**

Initially candidate questions from S2 Table were presented to 30 preoperative inguinal hernia patients and the relevance of each of the candidates with the hernia disease was rated with a five-point scale (from the most relevant to not relevant at all). Three questions, all from the operation-related domain, and the question of eventration, were considered irrelevant with a mean score of 3 (neutral response) or less. The investigators believed that the ambiguous nomenclature of seroma, hematoma, wound infection, and eventration was not easily recognized by hernia patients, and these complications could be readily detected by clinicians. As a consequence, these items were discarded.

S2 Table also showed the mapping relationships between the candidate questions and the proposed HERQL items. It’s not a simple one-to-one relationship; mutually exclusive candidates were stacked together (sex impairment, low back pain, gastro-intestinal symptoms, and micturition corresponding to Q10 of the HERQL) while the immediate pain severity and global outcome/treatment satisfaction questions were subdivided into more HERQL items to enhance sensitivity (Q01, Q03-Q05 for the immediate pain and Q18-Q20 for the global outcome/treatment satisfaction). In addition, interfering various activities, interfering walking or climbing stairs, and activities of daily life were summarized by Q07 and Q09, movement limitation by Q06 and Q09, and less accomplishment at home/work by Q06 and Q07 of the HERQL.

These 30 inguinal hernia patients were then asked to complete the preliminary version of the HERQL, with all Likert-type items scored by five-step rating scales. To further understand the underlying construct of the HERQL, successive explorative factor analyses were performed. Principal component analysis, which is a variable reduction technique, was performed and the first two components with eigenvalues more than unity accounted for 44% and 14% of the total variance, and these two components were retained, hinting that a smaller number of principal components could account for more than half of the response variation. Since quality of life measures were assumed to be reflective of the underlying constructs (domains), a common factor model was postulated with squared multiple correlations for the prior communality estimates.

An orthogonal varimax rotation followed by an oblique Procrustes rotation (PROMAX method) was adapted, and the first two factors were retained, which accounted for 101% of the common variance (62% and 39% for the first and second factor, respectively). The cumulative variance of the two retained factors might exceed 100% due to the non-orthogonal transformation and negative eigenvalues reported from five out of the ten common factors. The Scree test also supported the retention of two common factors (data not shown). S1 Fig. showed the graphical plot of PROMAX-rotated factor loadings. All symptomatic scales clustered together on the positive direction of the first factor, while the functional scales (Q11-Q13) clustered together on the negative direction. Among symptomatic scales, pain domain items (Q01, Q03-Q05) were intertwined with the activity restriction (Q09), while hernia protrusion (Q02) and analgesic usage (Q08), both of which were associated with a much smaller positive loading on the first factor, did show a substantial opposite loading on the second factor. The sample size of 30, however, is too limited to derive an interpretation of the second factor, but the bimodal distributions of symptomatic and functional items toward the opposite directions of the first common factor did support the symptom-function duality, paving the way for subsequent SEM in the filed study.
