# Supplementary material for: Quality of life of inguinal hernia patients in Taiwan: The application of the hernia-specific quality of life assessment instrument
Source: PLoS One. 2017 Aug 17;12(8):e0183138. doi: 10.1371/journal.pone.0183138 (PMC5560705; doi:10.1371/journal.pone.0183138)
Supplement: S2 File — (DOCX) [file pone.0183138.s006.docx]

**S2 File. Supplementary methods**

1. **EQ-5D-5L**

The EQ-5D-5L is a validated instrument for health outcomes measurement and the standard Taiwan Chinese version 1.0 was used after obtaining permission from the EuroQol group. Five dimensions including mobility, self-care, usual activities, pain/discomfort, and anxiety/depression were scored with five levels, from no problems to extreme problems.

1. **The unconditional linear growth model**

The mathematic formulas are:

*Y_ij_* = π*_0j_* _+_ π*_1j_* (TIME)*_ij_* _+_ γ*_ij_* , where

π*_0j_* = β*_00_* + μ*_0j_* and π*_1j_* = β*_10_* + μ*_1j_*

γ*_ij_* ～ *N*(0, ^2^) and (μ*_0j_* μ*_1j_*) ～ *N* [$\left( \begin{matrix} 0 \\ 0 \end{matrix} \right)$,$\left( \begin{matrix} 00 01 \\ 10 11 \end{matrix} \right)$]

*Y_ij_* represented the summative pain score of the *i^th^* survey of the *j^th^* patient, where *i* could be 0, 1, 3, indicating the pre-operative survey, immediately post-operative survey, and post-operative 3-month survey. In the mathematic formula, π is the within person parameter and β is the between-person parameter. The intercept (π*_0j_*) is the preoperative estimate of the initial value of the summative pain score, and the slope (π*_1j_*) is the estimate of the rate of change across repeated surveys. β*_00_* and β*_10_* is the fixed part of overall intercept and slope, while μ*_0j_* and μ*_1j_* is the accompanied random part, and *γ_ij_* is the within-person residual. The variance components, _00_ and _11_ represented variances of the intercept and slope, respectively, and _10_ (_01_) was the covariance between the intercept and slope. The variance of γ*_ij_* ^2^, was the variation among repeated measures within individual.

1. **Structure equation modeling (SEM)**

For SEM, model fitness assessed how well the proposed model captured the covariance between all the items of the questionnaire. Goodness of fit was evaluated by the ratio of chi-square to the degrees of freedom. A ratio less than three indicated a good fit of the hypothesized construct to the experimental data. Other fit indices included the goodness of fix index (GFI) > 0.90, adjusted GFI (AGFI) > 0.80, standardized root mean square residual (RMR) < 0.1, comparative fit index (CFI) > 0.9, and the root mean square error of approximation (RMSEA) < 0.08 [1-5]. For model identification purposes, all variances for latent factors were set to unity. Error variances of manifest variables (questionnaire items) and partial variances of endogenous latent variables were positively constrained. In the current study, only first-order latent factors were considered without further higher-order factors for conceptual interpretability.

Model modifications were performed according to the above fit index parameters and modification indices (Lagrange Multiplier tests) provided by SAS/STAT version 9.4 (SAS Institute Inc., Cary, NC) using the procedures CORR, NPAR1WAY, and CALIS. The default maximal likelihood (ML) method was used for parameter estimation. When the multivariate normality assumption was violated, the distribution-free diagonally weighted least square (DWLS) estimator was used. Correlations between latent variables at the same level were freely estimated.

**References**

1. Hu L, Bentler PM. Cutoff criteria for fit indexes in covariance structure analysis: conventional criteria versus new alternatives. SEM: AMJ. 1999;6: 1-55.
2. Bagozzi RP, Yi Y. **The degree of intention formation as a moderator of the attitude-behavior relationship.** Soc Psychol Q. 1989;52: 266-279.
3. Bentler PM, Bonett DG. Significance tests and goodness of fit in the analysis of covariance structures. Psychol Bull. 1980;88: 588-606.
4. Jarvenpaa, SL, Tractinsky N, Vitale M. Consumer trust in an internet Store. Inf Technol Manage. 2000;1: 45-71.
5. McDonald RP, Ho MR. Principles and practice in reporting structural equation analyses. Psychol Methods. 2002;7: 64-82.
