# Supplementary material for: Quality of life of inguinal hernia patients in Taiwan: The application of the hernia-specific quality of life assessment instrument
Source: PLoS One. 2017 Aug 17;12(8):e0183138. doi: 10.1371/journal.pone.0183138 (PMC5560705; doi:10.1371/journal.pone.0183138)
Supplement: S3 File — (PDF) [file pone.0183138.s007.pdf]

HERQL (Version 1.1)

* Required

Discomfort induced by hernia in the past week

1. **Please indicate the severity of pain induced by hernia at rest (i.e. lie down)** *

   Mark only one oval.

	0	1	2	3	4	5	6	7	8	9	10	
Not at all painful												Most severe pain imaginable

2. **Is there any protrusion or straining sensation over your abdominal wall/groin hernia?** *

   Mark only one oval.

   - Never
   - Seldom (once every several months)
   - Occasionally (several times a month)
   - Often (several times a week)
   - Persistent

3. **Please indicate the severity of pain induced by hernia during light activity (i.e. walking)** *

   Mark only one oval.

	0	1	2	3	4	5	6	7	8	9	10	
Not at all painful												Most sever pain imaginable

4. **Please indicate the severity of pain induced by hernia during moderate activity (i.e. climbing stairs)** *

   Mark only one oval.

	0	1	2	3	4	5	6	7	8	9	10	
Not at all painful												Most severe pain imaginable

5. **Please indicate the severity of pain induced by hernia during heavy activity (i.e. exercise or lifting)** *

Mark only one oval.

	0	1	2	3	4	5	6	7	8	9	10	
Not at all painful	○	○	○	○	○	○	○	○	○	○	○	Most severe pain imaginable

6. **Which conditions do you think are most affected by hernia?** *

(multiple selections)

Check all that apply.

- [ ] Not affected by hernia at all
- [ ] At rest
- [ ] Lifting heavy weights or defecation
- [ ] Long sitting, long standing, keep walking or change position
- [ ] Exercise

7. **Have you ever been restricted in certain activities due to hernia induced pain or discomfort?** *

(multiple selections)

Check all that apply.

- [ ] No restriction at all
- [ ] Daily activity (housework) restricted
- [ ] Outdoor activity (walking, bicycling, driving) restricted
- [ ] Exercise restricted
- [ ] Heavy labor restricted

8. **Have you ever taken analgesics due to hernia induced pain or discomfort?** *

Mark only one oval.

- ○ Never
- ○ Seldom (once every several months)
- ○ Occasionally (several times a month)
- ○ Often (several times a week)
- ○ Daily

9. **Please indicate the extent of activity restriction due to hernia induced pain or discomfort** *

Mark only one oval.

	0	1	2	3	4	5	6	7	8	9	10	
No restriction at all	○	○	○	○	○	○	○	○	○	○	○	Totally restricted

10. **Do you experience any discomfort in the past week?** *

(multiple selections)

Check all that apply.

- [ ] Not at all
- [ ] Upper gastrointestinal discomfort
- [ ] Lower gastrointestinal discomfort
- [ ] Back discomfort
- [ ] Urinary discomfort
- [ ] Sexual discomfort
- [ ] Other: ____________________

11. **Please descript the impact of hernia on your health** *

Mark only one oval.

- ( ) No impact at all
- ( ) Little impact
- ( ) Moderate impact
- ( ) Severe impact
- ( ) Extremely severe impact

12. **Does hernia elicit any economic burden for you?** *

Mark only one oval.

- ( ) No burden at all
- ( ) Little burden
- ( ) Moderate burden
- ( ) Severe burden
- ( ) Extremely severe burden

13. **Please value your global health/quality-of-life in the past week** *

Mark only one oval.

- ( ) Excellent
- ( ) Good
- ( ) Fair
- ( ) Poor
- ( ) Extremely poor

14. **Have you received surgical repair of hernia?** *

Mark only one oval.

- ( ) Not yet
- ( ) Repaired *Skip to question 15.*

Having received hernia repair surgery

15. **Have you ever experienced mesh foreign body sensation?**

(Skip this item if you did not receive mesh repair for hernia)
Mark only one oval.

- Never
- Seldom (once every several months)
- Occasionally (several times per month)
- Often (several times per week)
- Always

16. **Do you experience any discomfort associated with hernia surgery in the past week?** *

(multiple selections)
Check all that apply.

- [ ] Not at all
- [ ] Groin pain
- [ ] Groin or inner thigh paresthesia (numbness)
- [ ] Abdominal wall pain
- [ ] Abdominal wall paresthesia (numbness)
- [ ] Water contact or urination pain
- [ ] Coital pain
- [ ] Testicular pain
- [ ] Ovulation or menstration pain
- [ ] Other: ______________________

17. **Please indicate the severity of discomfort** *

Mark only one oval.

- No bother at all
- Little bothersome
- Moderate bothersome
- Very bothersome
- Extremely bothersome

18. **How do you rate your last hernia repair?** *

Mark only one oval.

- Very satisfactory
- Satisfactory
- Fair
- Unsatisfactory
- Very unsatisfactory

19. **Regarding outcome of hernia repair, you are....** *
Mark only one oval.

- ( ) Very confident that hernia will not recur
- ( ) Suppposely that hernia will not recur
- ( ) Have no idea whether hernia will recur
- ( ) Worried about that hernia may recur
- ( ) The hernia has recurred

20. **How do you rate the improvement in quality-of-life by hernia repair?** *
Mark only one oval.

- ( ) A lot of improvement in quality-of-life
- ( ) Slight improvement in quality-of-life
- ( ) No change in quality-of-life
- ( ) Slight deterioration in quality-of-life
- ( ) Devastating in quality-of-life

Powered by
Google Forms
